# Seasonality of physical activity and its association with socioeconomic and health factors among urban-dwelling adults of Kaunas, Lithuania

**DOI:** 10.1186/s12889-019-7399-4

**Published:** 2019-08-07

**Authors:** Audrius Dėdelė, Auksė Miškinytė, Sandra Andrušaitytė, Jolanta Nemaniūtė-Gužienė

**Affiliations:** Department of Environmental Sciences, Faculty of Natural Sciences, Vytautas Magnus University, Vileikos Street 8, 44404 Kaunas, Lithuania

**Keywords:** Physical activity, Walking, Cycling, Seasonality, Employment status, BMI

## Abstract

**Background:**

Physical activity (PA) has been declining dramatically over time in many countries worldwide. The decrease of PA levels affects a person’s health and quality of life as it is a significant risk factor for many noncommunicable diseases. Understanding the factors that determine PA is particularly important in promoting greater PA in adults and reducing the risk of diseases associated with physical inactivity. This study investigated associations of seasonal PA levels with socioeconomic and health factors among adults.

**Methods:**

A cross-sectional study included 1111 participants of Kaunas city, Lithuania who completed a questionnaire about PA and mobility behaviour, socioeconomic, health and demographic factors. Commuting PA and sufficient PA (sPA) on weekdays and weekends in the summer and winter seasons was investigated in this study. Data on daily commuting duration and forms of transportation were collected using a questionnaire survey. Daily commuting was categorized into two categories: 1) using motorized transportation or walking or cycling 0 to 29 min, 2) and walking or cycling for 30 min or more.

**Results:**

Our findings showed significant seasonal impact on PA levels. The results revealed that employment status was significantly associated with PA. Unemployed individuals were 2 times more likely to engage in sPA in winter and almost 3 times in summer compared to workers.

**Conclusions:**

Our findings suggest the importance of considering environmental, socioeconomic and health factors when assessing PA. Promoting PA through active commuting is an important part of a healthy lifestyle and strategies to support the implementation of health-promoting policies and practices are needed.

## Background

Physical activity (PA) has a health benefit that influences people’s quality of life [1, 2]. Researchers have proved that moderate- and vigorous-intensity PA improves health and contributes to prevent noncommunicable diseases [3–6]. According to the World Health Organization (WHO), globally, 1 in 4 adults is not active enough, more than 80% of the world’s adolescent population is insufficiently physically active and only one third of the EU inhabitants satisfy minimum recommendations of the WHO according to which adults aged 18–64 should do at least 150 min of moderate-intensity aerobic PA throughout a week [6, 7].

Global decline in PA is attributed to economic development with the subsequent increase in urbanization and communication technologies [8]. An increase in the use of passive modes of transportation, a lack of PA during leisure time and sedentary behaviour at work and home have been associated with declining PA levels [9, 10]. With economic growth, increasing number of people and vehicles in urban areas public lifestyle becomes less active.

Several environmental factors can discourage people from becoming more active. The influence of seasonality on PA and walking has been studied in mid˗latitude areas for younger adults [11]. Some studies have investigated the effects of seasonal changes in PA in the elderly [12]. Therefore, seasonality should be considered when analysing PA and walking [12, 13].

Previous studies have showed the association between PA and sociodemographic and socioeconomic factors [14, 15]. Beenackers et al. found that in studies reporting occupational PA, individuals in lower socioeconomic groups were more active, whereas the results were opposite for leisure-time PA, which showed that higher socioeconomic groups were more likely to be physically active in their leisure-time than lower socioeconomic groups [15]. Meanwhile, inconsistent results were found in terms of commuting PA and socioeconomic status [15]. There is also some evidence that full-time employment is positively associated with PA among men and job type has a major impact on PA levels in both genders [16]. Salmon et al. found that less-skilled workers, homemakers and those in lower status occupations were less likely to report leisure-time PA [17]. Other studies have reported opposite results with less-skilled and full-time workers as more active [18, 19]. Work-related PA has health benefits, but it is less beneficial than aerobic physical exercise during leisure time [20, 21]. Endurance capacity, which is particularly important with regard to preventing noncommunicable diseases, improves during aerobic leisure activities [22]. However, only high levels of PA during leisure time, as well as the increase in time spent in active commuting can actually compensate for negative effects of prolonged sitting, but these levels of leisure time PA are often not achieved [23, 24]. Mitsui et al. showed that male office workers in a rural area are more active on non-working days than on working days in the summer, with an inverse pattern in the winter season [25]. However, other studies conducted in urban areas have found different patterns, which showed that the level of PA is lower on non-working days than during working days and people’s participation in these activities are limited to professional work and daily living activities [26, 27]. These findings indicate that employment status is an important factor in assessing physical activity.

The aim of the present study was to investigate associations of environmental (seasonality), socioeconomic and health factors with PA levels among adults. The EU Commission actively promotes Sustainable Urban Mobility activities and supports the development and implementation of Sustainable Urban Mobility Plans (SUMPs) which provide guidelines for local authorities on how to improve the accessibility of urban areas and create cleaner and more sustainable transport modes to, through and within these areas. The assessment of the current urban mobility situation at the national level is necessary for the development and implementation of SUMP in each country. Lithuania lacks representative data on PA. Therefore, the results of our study have practical implications and are significant for all urban populations in Lithuania and, also, for urban populations in eastern European countries.

## Methods

### Study design and study population

A cross-sectional study with a random (single stage) sampling was conducted with Kaunas residents about their PA and mobility habits. A survey was carried out by a market research agency that has previously conducted similar surveys. The response rate for the survey was 57%. Males and females aged 18 years and over, who were permanent residents of the city and gave informed consent, were included in the study. Main outcome measure was self-reported commuting PA: walking and cycling. Participants were recruited and interviewed by telephone from September to November 2017. We included 1111 adults who completed the questionnaire. The survey was completely anonymous and no identifiable personal details were collected from the respondents.

The questionnaire included questions on demographic (age, gender, marital status, children), socioeconomic (educational level, employment, income, car disposal), health-related (body mass index (BMI), chronic disease, smoking habits, alcohol consumption) factors and PA. Questions were derived from existing and validated questionnaires, some of which were adapted for specific project objectives related to the effects of PA, sustainable mobility, seasonality on health. The Global Physical Activity Questionnaire developed by WHO was used to collect information about work and leisure time PA and travel behaviour [28].

#### Socioeconomic factors

Educational level was classified as low education (12 or fewer years), medium (non-university) and high education (university degree). According to the employment status, participants were classified into two groups: workers and unemployed individuals. Income was divided into two groups: lower- and middle-income (≤ 1000 Eur) and upper-income (> 1000 Eur). The income classification is based on a measure of income per person.

Participants were asked in the questionnaire if they had a car at their disposal to determine if car ownership is associated with less frequent active transport and less PA.

#### Health-related variables

In the questionnaire, participants were asked if they had been diagnosed with a chronic illness by a doctor to assess the prevalence of chronic disease. In order to calculate BMI, each participant was asked about his/her weight and height. BMI was calculated as the ratio of weight (kg) to height squared (m^2^). To assess the prevalence of obesity, it was defined as a BMI greater than or equal to 30.0 kg/m^2^. BMI was examined as a continuous variable (kg/m^2^) and as a categorical variable with two groups: non-obese (< 30 kg/m^2^) and obese (≥ 30 kg/m^2^).

Participants were classified into two smoking categories: current smokers and non-smokers. Alcohol consumption was examined as a categorical variable with two groups: non-drinkers and drinkers.

#### Demographic factors

Age was used as a categorical variable and divided into three groups: 1) ≤ 45 years old, 2) 46–64 years old and 3) ≥ 65 years old. Participants were classified into four marital status groups – married, divorced, single and widowed. Adults with children were analysed as a continuous variable and as a categorical variable with two groups: adults with one or more child under the age of 18 (< 18 y) were coded as yes and those adults who reported that they do not have a child or have one or more child over the age of 18 were coded as no.

#### Outcome

Commuting PA on weekdays and weekends during summer and winter were analysed. The respondents were asked whether they walked, rode a bicycle, or used motorized transportation to and from work or other frequently visited location as well as the daily duration of these activities. The daily commuting PA was categorized into two categories: using motorized transportation or walking or cycling 0 to 29 min; and walking or cycling for 30 min or more. Sufficient physical activity (sPA) (at least 150 min of PA per week) was assessed based on questions about the duration of daily physical activity.

### Study area

The city of Kaunas (54°56′ N, 24°51′ E; altitude 24–90 m) has a population of 292,691 (01-01-2017) distributed over a land area of 157 km^2^. Long-term (1981–2010) climate data are shown in Table 1.

**Table 1:** Climatic data in Kaunas

Characteristics	Mean value
Temperature, °C	
Mean (annual)	7.1–7.4
Mean (in summer)	13.8
Mean (in winter)	(−2)
Maximum (in summer: July)	18.0–18.1
Minimum (in winter: January–February)	(− 3.6) − (− 3.1)
Absolute minimum (in winter)	(− 31.2)
Absolute maximum (in summer)	35.1
Precipitation, mm	
Annual	600–640
In summer	≈ 335
In winter	≈ 305
Sunshine duration (annual), h	≈ 1870
Snow cover (in winter), days	65–80

### Statistical analysis

Participants’ characteristics combined and stratified by employment status in different seasons are presented as the mean and standard deviation (SD) or numbers and proportion. Statistical analyses in this study were conducted using the methods described by Jekel et al. [29] The dependent variable (the outcome) in this study was a categorical variable indicating the level of PA and sPA (150 min/week and more according to the WHO recommendations). The independent variables were demographic (age, gender, marital status, children), socioeconomic (employment status, educational level, income, car disposal) and health factors (BMI, chronic disease, smoking, alcohol consumption). Student’s *t*-test for independent groups (for continuous variables) and chi-square tests (for categorical variables) of the baseline characteristics were calculated during weekdays and weekends by employment status in the summer and winter.

Additionally, multivariable logistic regression was used to assess the association between the prevalence of sPA in the summer and winter and socioeconomic, health-related and demographic variables. Multiple independent variables such as employment status, educational level, income, car disposal, BMI (continuous variable), chronic disease, smoking exposure, alcohol consumption, age, gender, marital status, minor children (continuous variable) were entered into a model. The adjusted odds ratios (aORs) were presented of each independent variable after adjusting for the effects of other independent variables in the model. Statistical significance was set at *p* < 0.05. All statistical analyses were performed using SPSS version 25.0 (IBM Corp. released 2017. IBM SPSS Statistics for Windows, Version 25.0 Armonk, NY: IBM Corp).

### Ethics

The study received ethical approval from Kaunas Regional Biomedical Research Ethics Committee (Approval No. BE-2-16).

## Results

The distribution of individual variables between participants are shown in Table 2. The study included 1111 adults (57.7% were women and 42.3% were men). The average age of participants was 48.4 years (SD = 16.8). A total of 34.9% of participants had high educational level, 59.5% were employed at the current time. Smoking was reported by 30.0% of respondents. The prevalence of obesity was 16.9%.

**Table 2:** Characteristics of the study population (*N* = 1111)

Variable	N (%)
Gender	
Women	641 (57.7)
Men	470 (42.3)
Age group	
≤ 45	528 (47.5)
46–64	352 (31.7)
≥ 65	231 (20.8)
Age, mean (SD)	48.4 (16.8)
Educational level	
Low	416 (37.4)
Medium	307 (27.6)
High	388 (34.9)
Marital status	
Married	653 (58.8)
Divorced	131 (11.8)
Single	196 (17.6)
Widowed	131 (11.8)
Employment status	
Workers	661 (59.5)
Unemployed	450 (40.5)
Income (Eur) (*N* = 877)	
≤ 1000	545 (62.1)
> 1000	332 (37.9)
Car disposal	613 (55.2)
Smoking	333 (30.0)
Alcohol consumption	540 (48.6)
BMI (*N* = 903)	
< 30	750 (83.1)
≥ 30	153 (16.9)
BMI, mean (SD)	26.4 (4.7)
Children (< 18 y)	
No	772 (69.5)
Yes	339 (30.5)

According to the data of the Lithuanian Department of Statistics, the median age of the population of Kaunas in 2017, was 42 years, women comprised a larger proportion of the population compared to men, respectively 56.2 and 43.8%. Based on the data of Statistics Lithuania, the proportion of the entire population in each age group is: 43.1% (18–44 years), 32.1% (45–64 years) and 24.8% (≥ 65 years). The first two age groups are slightly different from the groups that were analysed in this study because the Lithuanian Department of Statistics only provides data for these specific age groups, however the age distribution of the entire population is similar to our sample.

The prevalence of PA levels from walking or cycling according to individual characteristics of participants stratified by employment status in summer are shown in Table 3. The results showed that the prevalence of higher levels of PA from walking or cycling (≥ 30 min/day) in summer was higher among unemployed individuals both on weekdays and weekends compared to workers. Among employed individuals, smokers and those having a car were found to have lower levels of PA both on weekdays and weekends (*p* < 0.05). Among unemployed participants who engaged in ≥30 min/day PA were observed lower mean values of BMI, lower prevalence of obesity and chronic disease compared to participants who engaged in lower levels of PA (< 30 min/day) in summer. Having a car was significantly associated with lower levels of PA among both workers and unemployed individuals on weekdays and weekends.

**Table 3:** The prevalence of PA levels from walking or cycling according to individual characteristics of participants on weekdays and weekends by employment status in summer

Physical activity	N (%)	Age, mean (SD)	BMI, mean (SD)	Obesity^a^ (%)	Education (%)			Smoking (%)	Chronic disease (%)	Income (%)		Car disposal (%)
					Low	Medium	High			≤1000	> 1000	
Workers												
Walking or cycling during weekdays												
< 30 min/day	634 (95.9)	42.0 (11.1)	25.9 (4.3)	12.7	27.6	28.4	44.0	35.5	19.6	41.3	58.7	74.0
≥ 30 min/day	27 (4.1)	43.2 (13.7)	24.4 (2.5)	5.9	22.2	29.6	48.1	14.8	14.8	50.0	50.0	51.9
*p* value		0.608	0.164	0.350	0.824			0.018	0.375	0.375		0.013
Walking or cycling during weekends												
< 30 min/day	630 (95.3)	42.1 (11.2)	25.9 (4.4)	12.8	27.8	28.1	44.1	35.7	19.7	41.4	58.6	74.0
≥ 30 min/day	31 (4.7)	41.9 (13.2)	24.3 (2.3)	4.5	19.4	35.5	45.2	12.9	12.9	46.7	53.3	54.8
*p* value		0.937	0.088	0.213	0.512			0.005	0.250	0.437		0.020
Unemployed												
Walking or cycling during weekdays												
< 30 min/day	367 (81.6)	57.8 (19.2)	27.4 (5.1)	24.8	53.1	26.7	20.2	23.2	61.9	92,1	7.9	33.2
≥ 30 min/day	83 (18.4)	58.1 (18.0)	25.2 (3.9)	10.3	48.2	25.3	26.5	22.9	51.8	86.7	13.3	9.6
*p* value		0.891	0.019	0.055	0.440			0.543	0.060	0.135		0.000
Walking or cycling during weekends												
< 30 min/day	366 (81.3)	57.8 (19.2)	27.4 (5.1)	24.9	53.3	26.8	19.9	23.2	62.0	92.1	7.9	32.8
≥ 30 min/day	84 (18.7)	57.8 (18.4)	25.2 (3.8)	10.0	47.6	25.0	27.4	22.6	51.2	86.7	13.3	11.9
*p* value		0.993	0.017	0.045	0.321			0.516	0.045	0.135		0.000

^a^ Obesity (BMI ≥ 30)

The prevalence of PA levels from walking or cycling according to individual characteristics of participants on weekdays and weekends by employment status in winter are shown in Table 4. The results showed that the prevalence of higher levels of PA in winter was higher among unemployed participants compared to workers. Similar trend was found during the summer. Higher prevalence of ≥30 min/day PA was found in unemployed individuals with higher income (18.6% vs 7.5%) on weekdays and weekends (*p* = 0.023) and in workers and unemployed individuals who did not have a car (*p* < 0.05).

**Table 4:** The prevalence of PA levels from walking or cycling according to individual characteristics of participants on weekdays and weekends by employment status in winter

Physical activity	N (%)	Age, mean (SD)	BMI, mean (SD)	Obesity^a^ (%)	Education (%)			Smoking (%)	Chronic disease (%)	Income (%)		Car disposal (%)
					Low	Medium	High			≤1000	> 1000	
Workers												
Walking or cycling during weekdays												
< 30 min/day	646 (97.7)	42.0 (11.2)	25.8 (4.3)	12.5	27.4	28.3	44.3	35.1	19.5	41.3	58.7	74.0
≥ 30 min/day	15 (2.3)	43.8 (13.7)	24.8 (3.2)	14.3	26.7	33.3	40.0	13.3	13.3	55.6	44.4	33.3
*p* value		0.545	0.714	0.609	0.908			0.063	0.420	0.298		0.001
Walking or cycling during weekends												
< 30 min/day	647 (97.9)	42.0 (11.2)	25.8 (4.3)	12.5	27.4	28.3	44.4	35.1	19.5	41.2	58.8	74.2
≥ 30 min/day	14 (2.1)	44.1 (13.8)	24.8 (3.2)	14.3	28.6	35.7	35.7	14.3	14.3	60.0	40.0	21.4
*p* value		0.499	0.533	0.609	0.777			0.086	0.471	0.191		0.000
Unemployed												
Walking or cycling during weekdays												
< 30 min/day	393 (87.3)	57.3 (19.4)	27.3 (5.7)	24.3	53,7	26.0	20.4	23.4	60.6	92.5	7.5	32.1
≥ 30 min/day	57 (12.7)	61.0 (16.1)	25.4 (3.5)	7.7	42.1	29.8	28.1	21.1	56.1	81.4	18.6	7.0
*p* value		0.114	0.174	0.145	0.229			0.419	0.310	0.023		0.000
Walking or cycling during weekends												
< 30 min/day	392 (87.1)	57.3 (19.3)	27.3 (5.1)	24.3	53.8	26.0	20.2	23.5	60.7	92.5	7.5	31.9
≥ 30 min/day	58 (12.9)	61.0 (16.8)	25.4 (3.5)	7.1	41.4	29.3	29.3	20.7	55.2	81.4	18.6	8.6
*p* value		0.167	0.155	0.117	0.158			0.389	0.253	0.023		0.000

^a^ Obesity (BMI ≥ 30)

Crude odds ratios and adjusted odds ratios (aOR) of socioeconomic, health-related and demographic characteristics associated with participation in sPA during winter and summer are shown in Table 5. We found that employment status was significantly associated with participation in sPA. Unemployed individuals were 3 times (aOR 3.14; 95% CI 1.11–8.87) more likely than workers to reach sPA in summer. A similar pattern of sPA by employment status was observed in the winter season (aOR 2.03; 95% CI 0.45–9.08), but there was no statistically significant difference between the two groups. We found that car disposal (aOR 0.07; 95% CI 0.01–0.64), (aOR 0.21; 95% CI 0.08–0.60) and the increase in BMI (aOR 0.85; 95% CI 0.74–0.98), (aOR 0.86; 95% CI 0.78–0.95) were significantly associated with lower odds of reaching sPA in both winter and summer seasons, respectively. We did not find any statistically significant association between educational level, gender, marital status and the participation in sPA.

**Table 5:** Associations between socioeconomic, health-related and demographic variables and the participation in sPA in winter and summer seasons

Characteristics	Winter		Summer	
	Crude OR (95% CI)	aOR^b^ (95% CI)	Crude OR (95% CI)	aOR^b^ (95% CI)
Socioeconomic variables				
Unemployed (vs workers)	5.72* (3.29–9.95)	2.03 (0.45–9.08)	4.50* (2.95–6.85)	3.14* (1.11–8.87)
Lower educational level (vs higher)	1.10 (0.68–1.77)	0.58 (0.19–1.74)	1.15 (0.78–1.69)	0.90 (0.41–2.01)
Income ≤1000 (vs > 1000)	2.10* (1.11–3.97)	2.76 (0.27–27.89)	2.34* (1.34–4.07)	1.28 (0.38–4.39)
Car disposal (vs no car disposal)	0.11* (0.06–0.21)	0.07* (0.01–0.64)	0.19* (0.12–0.30)	0.21* (0.08–0.60)
Health-related variables				
BMI^a^	0.96 (0.87–1.06)	0.85* (0.74–0.98)	0.92* (0,86–0.99)	0.86* (0.78–0.95)
Chronic disease (vs no chronic disease)	1.67* (1.05–2.67)	0.85 (0.21–3.40)	1.29 (0.88–1.90)	0.47 (0.18–1.26)
Smokers (vs non-smokers)	0.55* (0.31–0.99)	1.54 (0.35–6.79)	0.56* (0.35–0.89)	0.63 (0.21–1.96)
Alcohol consumption (vs no)	0.60* (0.37–0.96)	0.71 (0.19–2.71)	0.61* (0.41–0.89)	0.59 (0.23–1.49)
Demographic variables				
Age group				
46–64 (vs ≤45)	1.23 (0.64–2.36)	2.81 (0.50–16.22)	1.15 (0.71–1.87)	1.76 (0.55–5.63)
≥ 65 (vs ≤45)	4.75* (2.72–8.31)	3.02 (0.39–23.22)	3.03* (1.93–4.77)	1.66 (0.44–6.28)
Women (vs men)	1.54 (0.94–2.52)	0.85 (0.22–3.31)	0.98 (0.66–1.43)	0.45 (0.19–1.08)
Single (vs married)	1.38 (0.87–2.21)	1.26 (0.39–4.06)	1.16 (0.79–1.71)	0.53 (0.23–1.21)
Children (< 18 y)^a^	0.61* (0.41–0.90)	2.12 (0.74–6.08)	0.57* (0.41–0.79)	0.96 (0.43–2.14)

^a^ continuous variable

^b^ each independent variable is adjusted for all the other independent variables

^*^p < 0.05

## Discussion

We examined seasonal differences in physical activity levels among workers and unemployed individuals and associations between sPA and individual characteristics. Our results showed that unemployed individuals were 2 times more likely to engage in sPA in winter and almost 3 times in summer compared to workers. The percentage of adults who engaged in sPA were higher in the summer than in the winter among both workers and unemployed individuals. Previous studies have found similar results that unemployed individuals are more likely to reach recommended PA levels compared to workers [19, 30]. The relationship between retirement and higher levels of leisure-time PA was found in these studies. Meanwhile, other studies found the association between retirement and a greater volume of PA, but there was no difference in achieving the recommended 150 min/week of PA between retired and employed adults [31]. Our findings related to seasonal patterns of PA were similar to some previous studies in older adults in Reykjavik [32], in the UK [33], in Canada [34], in the U.S. [14], where the levels of PA were higher during the summer than during the winter.

Workers were slightly more physically active on weekdays than on weekends in winter and vice versa in summer. Meanwhile, unemployed participants were more likely to engage in sPA on weekends in both seasons. The differences in sPA among workers and unemployed individuals were higher in winter than in summer. Previous studies on weekday-weekend patterns of physical activity in adults showed similar PA levels between weekdays and weekends [35, 36]. However, a complex set of factors such as day type, season and employment status have not been taken into account when assessing PA in previous studies.

Many studies indicate an inverse relationship between PA and BMI [37, 38]. Flint et al. found that individuals who commuted to work by active and public transport modes had significantly lower BMI and percentage body fat than those who used private transport [37]. The results from our study showed that BMI and the prevalence of obesity were lower in individuals who engaged in ≥30 min/day of PA among both workers and unemployed individuals. Statistically significant difference in BMI between two PA groups were found for unemployed individuals in summer. Overall, the results showed that higher rates of obesity were observed among unemployed individuals for both PA groups in summer and for those of lower PA group in winter compared to workers. The study conducted by Cooper et al. found that obese participants (BMI > 30) were significantly less active than non-obese participants [39]. A population-based study carried out in Norway found similar results that overweight and obese participants engaged in less PA compared with normal weight individuals and these differences were most pronounced on the weekend [40].

Prevalence of sPA in summer and winter was higher among workers with high educational level and among unemployed individuals with low educational level compared to other groups. Martínez-González et al. found a significant trend to higher leisure time activity in participants with higher educational levels [41]. Opposite results were obtained from a study in Netherlands which found that there were no significant differences in PA between educational levels among adults [42].

The association between smoking and PA has been analysed in this study. Smoking was more prevalent in adults engaging in lower levels of PA among both workers and unemployed individuals, and the difference was more pronounced among workers. Our findings support previous research which showed that smoking was associated with physical inactivity and smokers tended to exercise less, compared to non-smokers [43].

Physical inactivity is a leading cause of most chronic diseases [44]. The prevalence of chronic disease was higher among workers and unemployed individuals with lower levels of PA in summer and winter. Previous studies have found similar associations between chronic disease and the levels of PA [45, 46]. The study conducted in 11 European countries found that physically inactive adults had a higher prevalence of chronic diseases and chronic disease symptoms compared with physically active adults [46].

Workers with higher incomes were less likely to reach ≥30 min/day of PA than those with lower incomes in both seasons. The opposite results were found among unemployed individuals, those with higher incomes were more physically active than those with lower incomes. The previous literature suggested that income is strongly associated with PA [47–49]. Shuval et al. examined the relationship between income and intensity of PA among U.S. adults and assessed that higher annual household income is related to more intense, less frequent patterns of PA and more daily sedentary time [49]. A study by Kari et al. indicated that higher income was associated with higher self-reported PA for both genders, while the pedometer-based results were gender-specific and the association was positive only for women [47]. Kim et al. study in Korea showed that higher incomes were associated with continuous increase in PA [48].

We found that car ownership was associated with less PA among workers and unemployed individuals for both seasons. These results are consistent with previous studies that found a negative relationship between car ownership and PA [50, 51].

Our findings showed statistically significant associations of sPA with employment status, car disposal and BMI. Higher odds of reaching sPA were assessed in individuals who reported not having a car and those with lower BMI in both seasons and in unemployed individuals in summer when controlling for confounders. A study by Mein et al. showed that the odds of reporting recommended PA levels were higher among part time workers, or those who had retired [19]. These results suggest that full-time workers have reduced opportunities for PA [52, 53].

This study has advantages over previous studies because the country’s geographical position allows a good assessment of physical activity patterns by seasonality, and the results of the study can be applied to the eastern and northern European countries. The data at the individual level allow to control for all covariates associated with physical activity, such as gender, age, educational level, employment status, car disposal, BMI, smoking and others. Using individual data, we addressed possible confounding variables in multiple regression analyses and estimated the association between individual characteristics and participation in sPA in winter and summer.

The EU Commission actively promotes Sustainable Urban Mobility Planning. Guidelines were developed, which provide local authorities with a framework for the development and implementation of these plans. However, for promoting those practices at their national level Member States need local data. The results of this study are actual for all urban populations in Lithuania and in eastern European countries. The analysis of national SUMP programmes was conducted at European, national and local levels by both CIVITAS SUMPs-Up and PROSPERITY projects [54]. The analysis showed that even experienced SUMP cities need support in areas such as transport evaluation and newer mobility policy areas [54]. The results of the present study can be used to assist SUMPs planners to tackle public health issues related to active mobility.

Some limitations of the present study should be noted. First, self-reported measures of PA have been used in the current study that could lead to overestimation or underestimation of individuals’ actual levels of PA. However, self-reported questionnaires are commonly used for the evaluation of PA in epidemiological and clinical studies, because of their low cost, they are easy to use and administer, they do not require as much time as direct measures and can measure different types of activities. The second limitation of this study is relatively small sample size that was limited in some subgroups, especially the number of individuals who participate in sPA, which could reduce the effects of individual characteristics on PA. Also, due to the nature of the cross-sectional study design, it is difficult to derive causal relationships. However, the small sample size did not negate significance of our findings as the study population was representative to the entire population of Kaunas city. Future work will examine self-reported and objectively (accelerometer) measured levels of PA in individuals.

## Conclusions

The association of PA levels with socioeconomic and health-related factors among workers and unemployed individuals were investigated considering seasonal differences. Our findings suggest that car ownership and the increase in BMI are associated with reduced odds of reaching sPA levels in both seasons. Unemployed individuals were more likely to engage in sPA in both the summer and the winter seasons. Our study suggests the importance of considering socioeconomic and health-related characteristics when assessing PA levels. Seasonal analysis also revealed different patterns in the levels of PA. The results of this study will enhance and make local and regional SUMPs as well as their revised versions more comprehensive. The increased awareness of environmental and livability issues in urban mobility already highlights the change in the perception of the role of mobility, which efficiency is no longer seen as a stand-alone goal but rather as a mean to achieve the objectives of a more comprehensive urban policy, including environment, sustainability, social dimension, safety and health. Better knowledge of PA issues and mobility depending on seasonality could supplement and orient SUMPs towards real positive health effects in urban environment.

## Data Availability

Requests for the transcripts should be addressed to the corresponding author.

## Abbreviations

aOR: Adjusted Odds Ratios
BMI: Body Mass Index
CI: Confidence Interval
PA: Physical activity
SD: Standard Deviation
sPA: Sufficient Physical Activity
SUMP: Sustainable Urban Mobility Plan
WHO: World Health Organization
